# Cuproptosis-Related genes in the prognosis of colorectal cancer and their correlation with the tumor microenvironment

**DOI:** 10.3389/fgene.2022.984158

**Published:** 2022-09-28

**Authors:** Weiqiang Wu, Jingqing Dong, Yang Lv, Dongmin Chang

**Affiliations:** ^1^ Department of Surgical Oncology, The First Affiliated Hospital of Xi’an Jiaotong University, Xi’an, Shaanxi, China; ^2^ Department of Ophthalmology, The 940th Hospital of Joint Logistics Support Force of Chinese PLA, Lanzhou, China; ^3^ Department of General Surgery, Guangzhou Red Cross Hospital, Medical College, Jinan University, Guangzhou, China

**Keywords:** colorectal cancer, cuproptosis, overall survival, colorectal cancer, immune

## Abstract

Colorectal cancer (CRC) is a common tumor disease of the digestive system with high incidence and mortality. Cuproptosis has recently been found to be a new form of cell death. The clinical significance of cuproptosis-related genes (CRGs) in CRC is not clear. In this study, The Cancer Genome Atlas Colon and Rectal Cancer dataset was used to analyze the relationship between CRGs and clinical characteristics of CRC by differential expression analysis and Kaplan–Meier survival (K-M) analysis. Based on CRGs, prognosis model and risk score of CRC was constructed in COADREAD by multivariate Cox analysis. Receiver operating curves (ROC) analysis, K-M analysis and calibration analysis in GDC TCGA Colon Cancer dataset were applied to validating model. Subsequently, the relationship between risk score of CRC and immune microenvironment was analyzed by multiple immune score algorithms. Finally, we found that most CRGs were differentially expressed between tumors and normal tissues. Some CRGs were differentially expressed among different clinical characteristics. K-M analysis showed that the CRGs were related to overall survival (OS), disease-specific survival, and progression-free survival. Subsequently, DLAT and CDKN2A were identified as risk factors for OS in CRC by multivariate Cox analysis, and the risk score was established. K–M analysis showed that there was a significant difference in OS between the high-risk and low-risk groups, which were grouped by risk score median. ROC analysis showed that the risk score performs well in predicting the 1-year, 3-year and 5-year OS. Enrichment analysis showed that the differentially expressed genes between the high- and low-risk groups were enriched in immune-related signaling pathways. Further analysis showed that there were significant differences in the levels of immune cells and stromal cells between the high- and low-risk groups. The high-risk group had higher levels of immune cells and interstitial cells. At the same time, the high-risk group had a higher immune escape ability, and the predicted immune treatment response in the high-risk group was poor. In conclusion, CRGs can be used as prognostic factors in CRC and are closely related to the levels of immune cells and stromal cells in the tumor microenvironment.

## Introduction

Colorectal cancer (CRC) is a common tumor disease of the digestive system (Jo and Oh, 2019). CRC can be cured by radical surgery combined with radiotherapy or radiotherapy (Zhong et al., 2022). However, most patients with CRC are in an advanced stage at the time of discovery, and tumor metastasis is still one of the main causes of mortality (Xi and Xu, 2021). In addition, although tumor pathological staging has important value in predicting prognosis, in view of the high incidence and mortality of CRC, more effective prognostic models need to be developed.

Copper is one of the necessary trace elements for the human body. It was found that the copper level in tumors was significantly higher than that in adjacent tissues (Ishida et al., 2013). This indicates that copper plays an important role in the growth of tumor cells, and the imbalance of copper homeostasis can lead to cytotoxicity and affect the occurrence and development of tumors (Ge et al., 2022). A recent study found that a new type of cell death was mediated by copper, namely, cuproptosis (Tsvetkov et al., 2022). Copper interacts with lipoylated members of the tricarboxylic acid cycle (TAC), thereby causing protein stress and eventually cell death (Tsvetkov et al., 2022). These genes that interact with copper in the tricarboxylic acid cycle are called cuproptosis-related genes (CRGs). It has been shown that the tricarboxylic acid cycle in CRC is reprogrammed to reduce energy production so that tumor cells can continue to survive under nutrient deficiency (Neitzel et al., 2020). Reprogrammed TCA has important role in CRC development and GRGs in TCAs were key candidate genes for CRC development. In addition, immune escape, as an important feature of tumors, protects tumor cells from immune cell attack (Engelsen et al., 2022). TCA-derived metabolites in tumor have multiple positive roles in supporting escape of immune cells (Scagliola et al., 2020). In view of the high concentration of copper in tumor tissues and the reprogramming of the TCA, CRGs will hopefully be used to develop new prognostic models for CRC.

In our study, we performed differential expression analysis to identify expression of CRGs in CRC and found that CRG expression was widely dysregulated in CRC. Next, survival analysis of CRGs in CRC were performed to detect the relationship between CRGs and prognosis in CRC and it was found that CRGs was related to tumor prognostic indicators, including overall survival (OS), disease-specific survival (DSS) and progression free interval (PFI). Subsequently, a prognostic risk model based on CRGs was developed that had a significant relationship with OS. In addition, immune-related scores were investigated to identify the relationship between CRGs and immune microenvironment and it was found that there were significant differences in intratumor interstitial cells between the high-risk and low-risk groups. We aimed to emphasize the importance of CRGs in the development of CRC and the close relationship between CRGs and intratumor interstitial cells.

## Materials and methods

### Data source

The Cancer Genome Atlas (TCGA) Colon and Rectal Cancer (COADREAD) dataset and GDC TCGA Colon Cancer (COAD) dataset was downloaded from the UCSC Xena server. TCGA, a landmark cancer genomics program based on American population, molecularly characterized over 20,000 primary cancer and matched normal samples spanning 33 cancer types. TCGA was built by National Cancer Institute and National Human Genome Research Institute. COADREAD in UCSC Xena server was derived from TCGA Data coordinating Center in January 2016 and had 434 samples. COAD data in UCSC Xena server was derived from TCGA data coordinating Center in August 2019 and had 512 samples. Gene expression in COADREAD and COAD was converted to log2 (1 + counts). COADREAD data were used as the training set in this study. After removing the same samples from training set, the remaining samples in COAD were used as the validation set.

### Principal component analysis

Principal component analysis (PCA) methods (Trozzi et al., 2021) were used to evaluate gene expression patterns in cancer samples and normal samples in training set.

### Construction and validation of a signature based on cuproptosis-related genes

In the training set, multivariate Cox regression with forward stepwise selection was used to identify CRGs related to OS. The cuproptosis-related prognostic risk score (CPRS) of each sample was calculated using the formula CPRS = Σ Exp (mRNAί) × Coefficient (mRNAί). All tumor samples were grouped by the CPRS median and named the low-risk group and the high-risk group. Kaplan–Meier survival analysis and time-dependent receiver operating characteristic (ROC) curves between the low-risk and high-risk groups were used to evaluate the prognostic predictive performance in training sets. Furthermore, survival analysis and time-dependent ROC analysis were also used in the validation set (R package: timeROC). The independent predictive variables identified by multivariate Cox regression analyses were used to construct the predictive nomogram and the corresponding calibration curves (R package: rms) in training set.

### Differential expression analysis

Differentially expressed genes (DEGs) between the low-risk and high-risk groups were identified with the limma (version 3.40.6) package in training set (Ritchie et al., 2015). Genes that met the criteria of *p* < 0.05 and log2Fold change >1.5 were regarded as DEGs between the two groups.

### Enrichment analysis

GO and KEGG enrichment analyses of DEGs in training set were performed by clusterProfiler (version 3.14.3) (Yu et al., 2012). The minimum gene set was set as 5, and the maximum gene set was set as 5000. A *p* value <0.05 and an FDR <0.01 were considered statistically significant.

### Investigation of immune-related scores

ESTIMATE analysis was employed to estimate the proportion of immune cells and stromal cells in the tumor tissue in training set. MCP-Counter analysis was employed to further estimate human immune and mesenchymal cell subsets according to the gene expression data in training set. The TIDE score and predicted response to immunotherapy were obtained via the TIDE database (http://tide.dfci.harvard.edu/) in training set.

### Immunohistochemical image source and quantification of immunostaining

Immunohistochemical images of CRGs in CRC and normal colorectal tissues were downloaded from the Human Protein Atlas (https://www.proteinatlas.org/). The H-score was used to quantify the staining.

### Statistical analysis

All statistical analyses and visualizations were carried out by R version 4.0.2 (http://www.r-project.org). For detecting CRG differential expression, the Wilcoxon signed-rank test was used to estimate the differences between normal and tumor groups, and the Kruskal–Wallis test was used to compare more than two groups in different clinical characteristics. Also, ESTIMATE score, MCP-Counter score and TIDE score were compared between low and high risk groups by Wilcoxon signed-rank test. For identifying relationship between CRG and CRC prognosis, Kaplan-Meier analyses and the log-rank test were used to assess the survival differences between low and high levels of CRGs. Prognosis between high and low risk groups were compared by Kaplan-Meier analyses and the log-rank test. Spearman analysis was used to compute the correlation coefficients between immune score and DLAT/CDKN2A. *p* < 0.05 was regarded as statistically significant.

## Results

### Expression patterns of CRGs in different clinical characteristics of CRC

**FIGURE 1:**
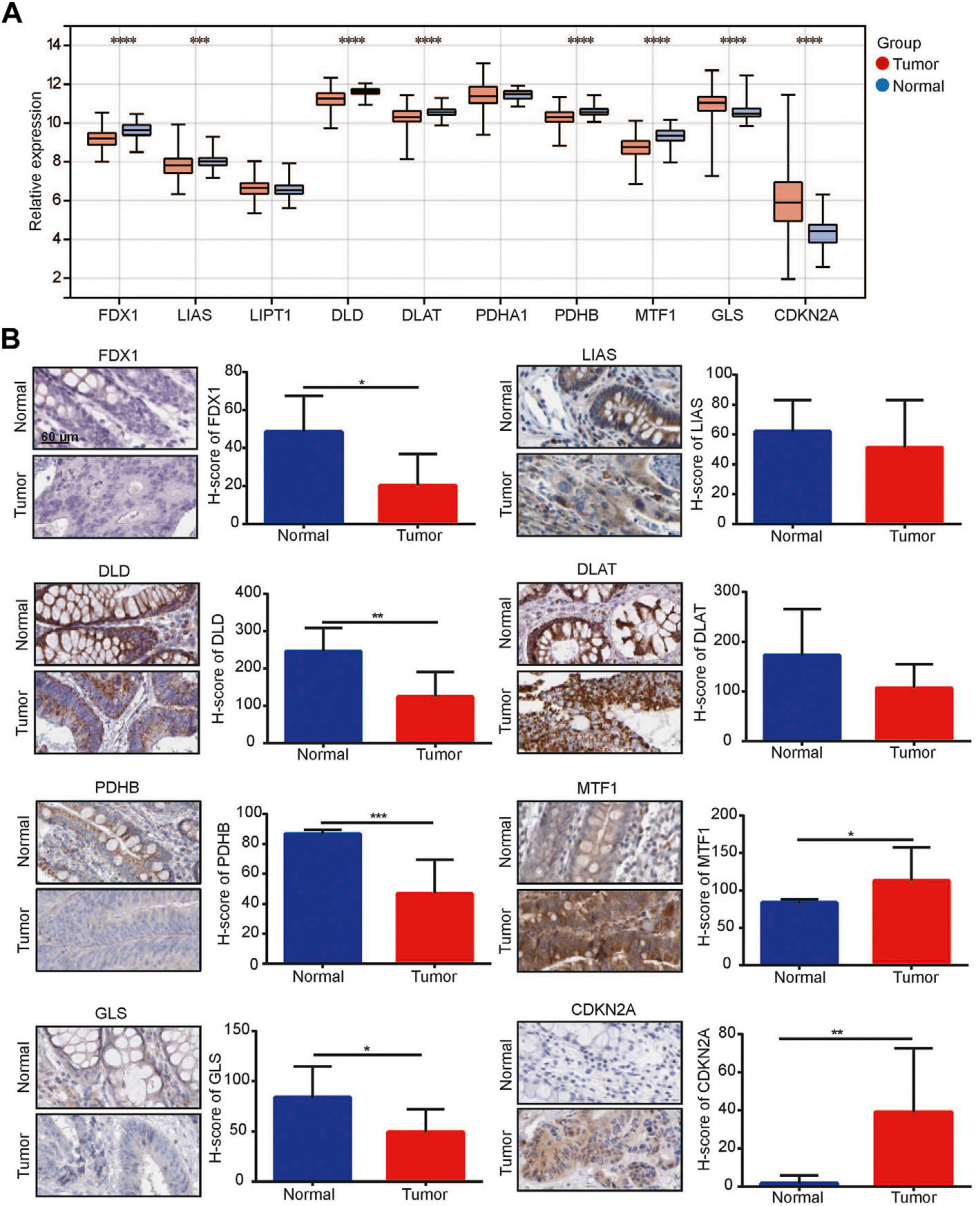
^1^Cuproptosis-related genes (CRGs) was related to colorectal cancer. **(A)** Expression of CRGs in TCGA-colorectal cancer dataset; **(B)** Immunohistochemical analysis for CRG expression between normal colon/rectal tissue and colorectal cancer. *p < 0.05, **p < 0.01, ***p < 0.001, ****p < 0.0001.

We obtained all CRGs including FDX1, LIAS, LIPT1, DLD, DLAT, PHDA1, PDHB, MTF1, GLS and CDKN2A from previous literature (Tsvetkov et al., 2022). We performed PCA on the TCGA CRC dataset, and one sample in the tumor group was removed (Supplementary Figure S1A). Subsequently, we detected the expression of these CRGs in tumor and normal samples and found that 6 genes, FDX1, LIAS, DLD, DLAT, PDHB and MTF1, were significantly downregulated in tumors, while 2 genes, GLS and CDKN2A, were significantly upregulated (Figure 1A). Immunohistochemical staining showed that among these CRGs with *p* < 0.05 in TCGA, their protein expression trend was consistent with the RNA expression in TCGA (Figure 1B).

**FIGURE 2:**
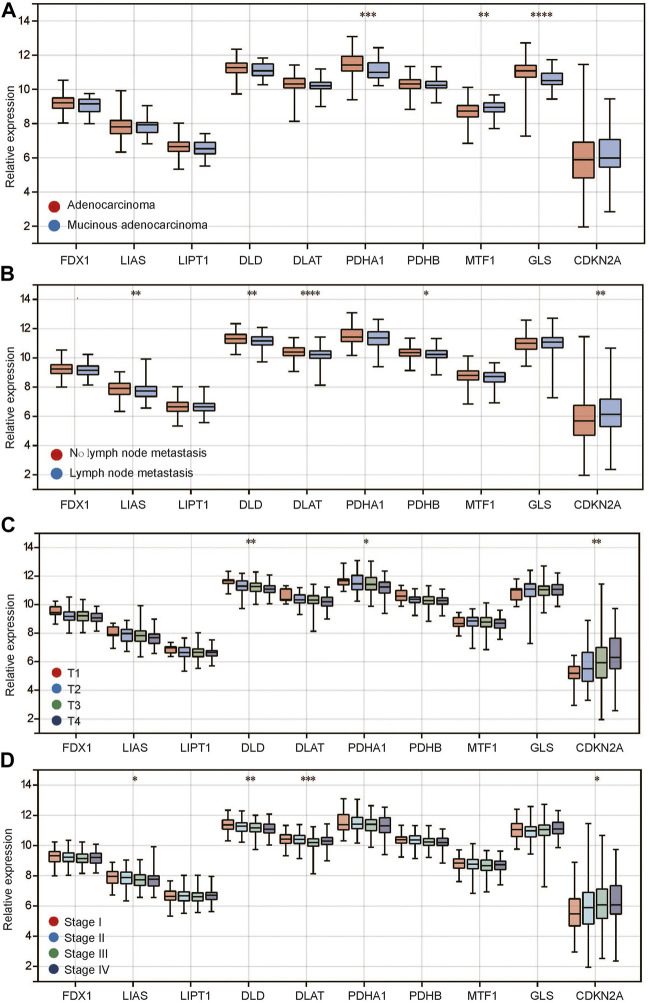
Relationship between CRG expression and clinical characteristics of colorectal cancer. **(A)** CRG expression in different histopathological types; **(B)** CRG expression in different status of lymph node metastasis; **(C)** CRG expression in different T stages; **(D)** CRG expression in different pathological stages. *p < 0.05, **p < 0.01, ***p < 0.001, ****p < 0.0001.

In tumors, we found that compared with adenocarcinoma, PDHA1 and GLS were expressed at low levels in mucinous adenocarcinoma, while CDKN2A was highly expressed (Figure 2A). In addition, between the recurrent and nonrecurrent tumor samples, only LIAS expression was different (Supplementary Figure S1B). Meanwhile, there was no difference in any CRGs between M0 and M1 (Supplementary Figure S1C), indicating that these genes may not be related to distant metastasis of the tumor. In terms of lymphatic metastasis, we found that 5 genes, including LIAS, DLD, DLAT, PDHB and CDKN2A, had significantly different expression between tumors with and without lymphatic metastasis (Figure 2B). LIAS, DLD, DLAT and PDHB were expressed at low levels in the group with lymphatic metastasis, while CDKN2A was highly expressed. Regarding T stage, we found that the expression of most genes showed a positive or negative trend with the T stage (Figure 2C). Among them, DLD, PDHA1 and CDKN2A were significantly differentially expressed among different T stages. DLD and PDHA1 expression decreased gradually with T stage, while CDKN2A expression increased gradually. In addition, we observed a similar phenomenon for stage. The expression of LIAS, DLD, DLAT and CDKN2A decreased with stage (Figure 2), while the expression of CDKN2A increased. Different tumor pathological types have different molecular expression characteristics. Therefore, we divided CRC into adenocarcinoma and mucinous adenocarcinoma and then observed the expression of CRGs among different clinical features. The results showed that the expression of CRGs (Supplementary Figures S2A–C) in adenocarcinomas was similar to that before in different clinical features including lymphatic metastasis, T stage and stage (Figures 2B–D). However, there were no differences in different clinical features in mucinous adenocarcinoma (Supplementary Figures S2D–F). The above studies suggest that there is a close relationship between CRG expression and the clinical characteristics of CRC.

### Relationship between CRGs and survival in CRC

**FIGURE 3:**
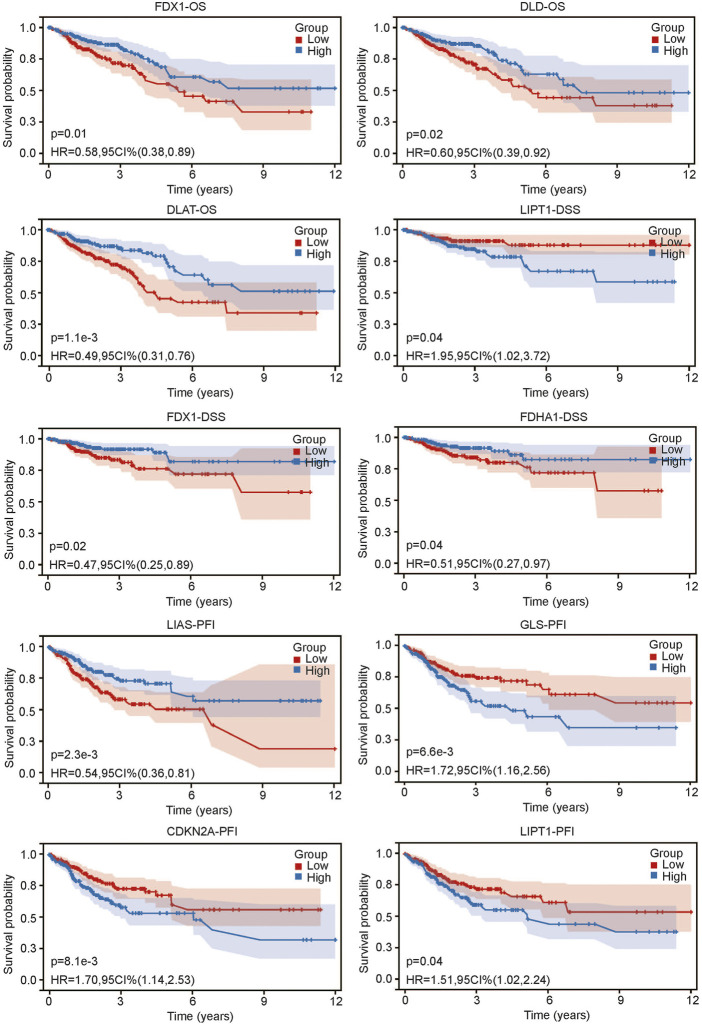
Relationship between CRG expression and prognosis of colorectal cancer including overall survival (OS), disease specific survival and progress free interval.

After we found that CRG expression was closely related to the clinical characteristics of CRC, we sought to determine whether CRG was related to prognosis. We grouped CRC patients according to the median expression value of each gene. The results showed that not all CRGs were related to prognosis. Three genes, PDX1, DLD1 and DLAT, were associated with OS (all *p* < 0.05) (Figure 3). The lower expression of these genes was associated with worse OS. Three genes, LIPT1, FDX1 and PDHA1, were related to DSS (all *p* < 0.05) (Figure 3). Higher LIPT1 expression was associated with worse DSS, while lower PDX1 and PDHA1 expression was associated with worse DSS. Additionally, 4 genes, LIAS, GLS, CDKN2A and LIPT1, were related to PFI (Figure 3). The lower expression of LIAS was associated with higher PFI, and the lower expression of GLS, CDKN2A and LIPT1 was associated with worse PFI. The above results suggest that there is a certain relationship between CRG expression and prognosis in CRC.

### Construction of a prognostic signature and nomogram of CRGs in CRC

**FIGURE 4:**
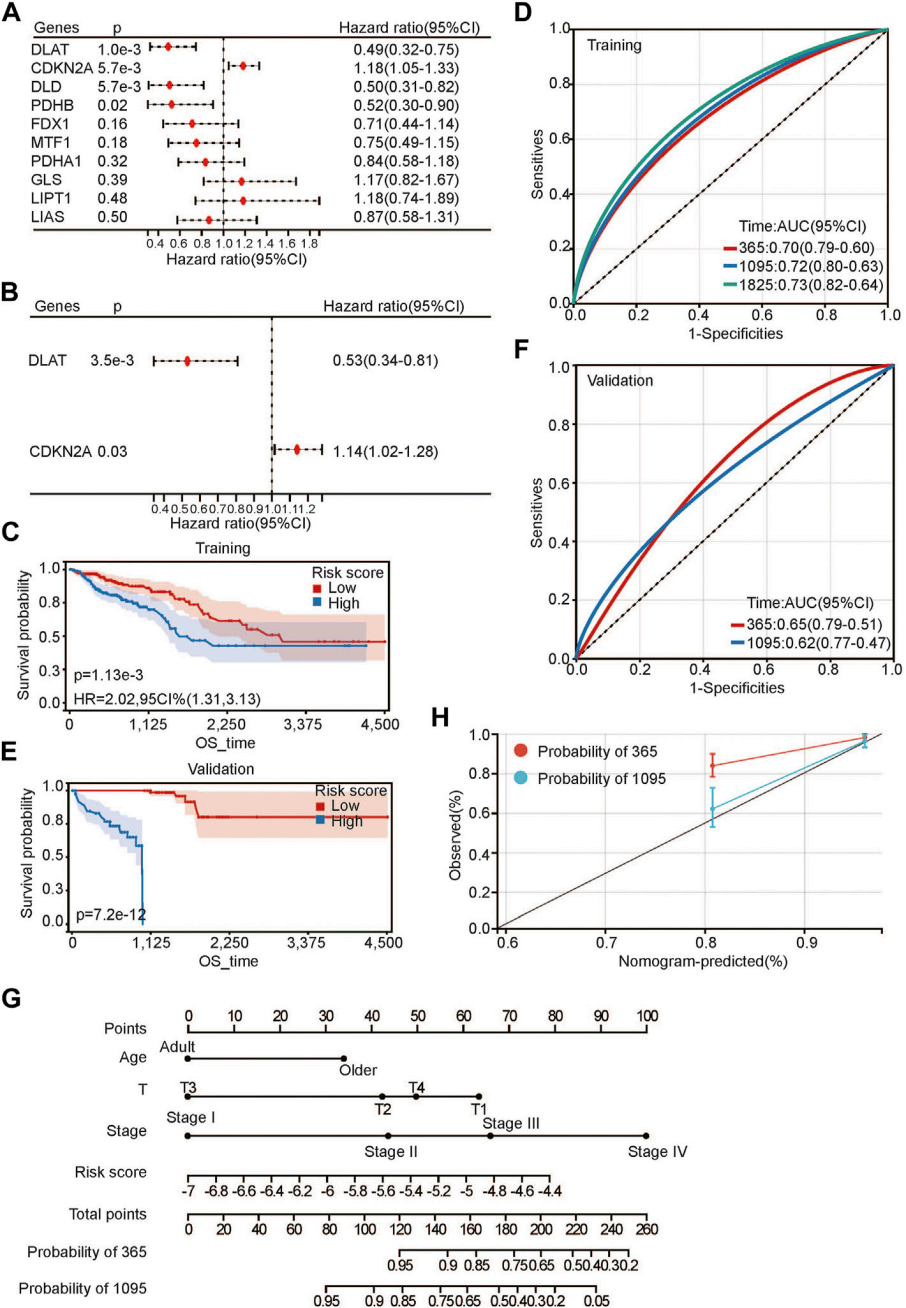
Developing prognosis risk score based on CRGs. **(A**,**B)** univariate **(A)** and multivariate **(B)** Cox regression analysis of CRGs for OS; **(C**,**E)** Kaplan-Meier analysis of risk score for OS in training set **(C)** and validation set **(E)**;**(D**,**F)** ROC analysis of risk score for OS in training set **(D)** and validation set **(F)**; **(G)** Nomogram construction of OS based on risk score and clinical characteristics; **(H)** Calibration curve analysis for constructed nomogram.

To further evaluate the relationship between CRG and prognosis, we first performed univariate Cox regression analysis of CRGs for OS. DLAT, CDKN2A, DLD, and PDHB were highly correlated with OS (Figure 4A). Then, multivariate Cox regression analysis was performed to further evaluate the relationship. The results showed that only DLAT and CDKN2A had a significant relationship with OS (Figure 4B). We believe that these two genes are most closely related to the prognosis of CRC. Subsequently, we used these two genes to construct a prognostic risk score using their regression coefficients: score = −0.641310371 × DLAT +0.130766422 × CDKN2A. Kaplan–Meier survival analysis showed that higher risk scores were associated with poorer OS (Figure 4C). With the increase in risk score, the survival rate decreased significantly. As a protective factor, DLAT expression decreased gradually as the risk score increased, and the expression level of CDKN2A increased gradually as a risk factor (Supplementary Figure S1D). The prediction accuracy of the area under the curve (AUC) assessment for 1 year, 3 years and 5 years were all more than 70% (Figure 4D). To further evaluate the prognostic risk model, we calculated the risk scores of each sample in the validation set. After the samples were divided into high-risk and low-risk groups according to the median score, the results were consistent with the previous results in the training set. Patients with high risk scores had a worse prognosis (Figure 4E). The ROC curve showed that the prediction accuracies at 1 year and 3 years were 0.65 and 0.62, respectively (Figure 4F). The above results suggested that the risk score we constructed is robust for predicting the prognosis of CRC patients.

Next, we conducted Cox multivariate analysis with forward stepwise selection of clinical characteristics and the risk score for OS. Age (young represented age<65 and older represented age≥65), T stage, pathological stage and risk score were identified as risk factors for the prognosis of CRC (Supplementary Table S1). Then, we built a nomogram for visualization (Figure 4G). C-index was calculated to be 3.59494602157775e-24 for OS, suggesting a relatively excellent predictive performance of the nomogram. Additionally, calibration plots demonstrated favorable concordance between the predicted OS and the observed OS at 1 and 3 years (Figure 4H).

### Correlation between interstitial cells and risk score-based CRGs

**FIGURE 5:**
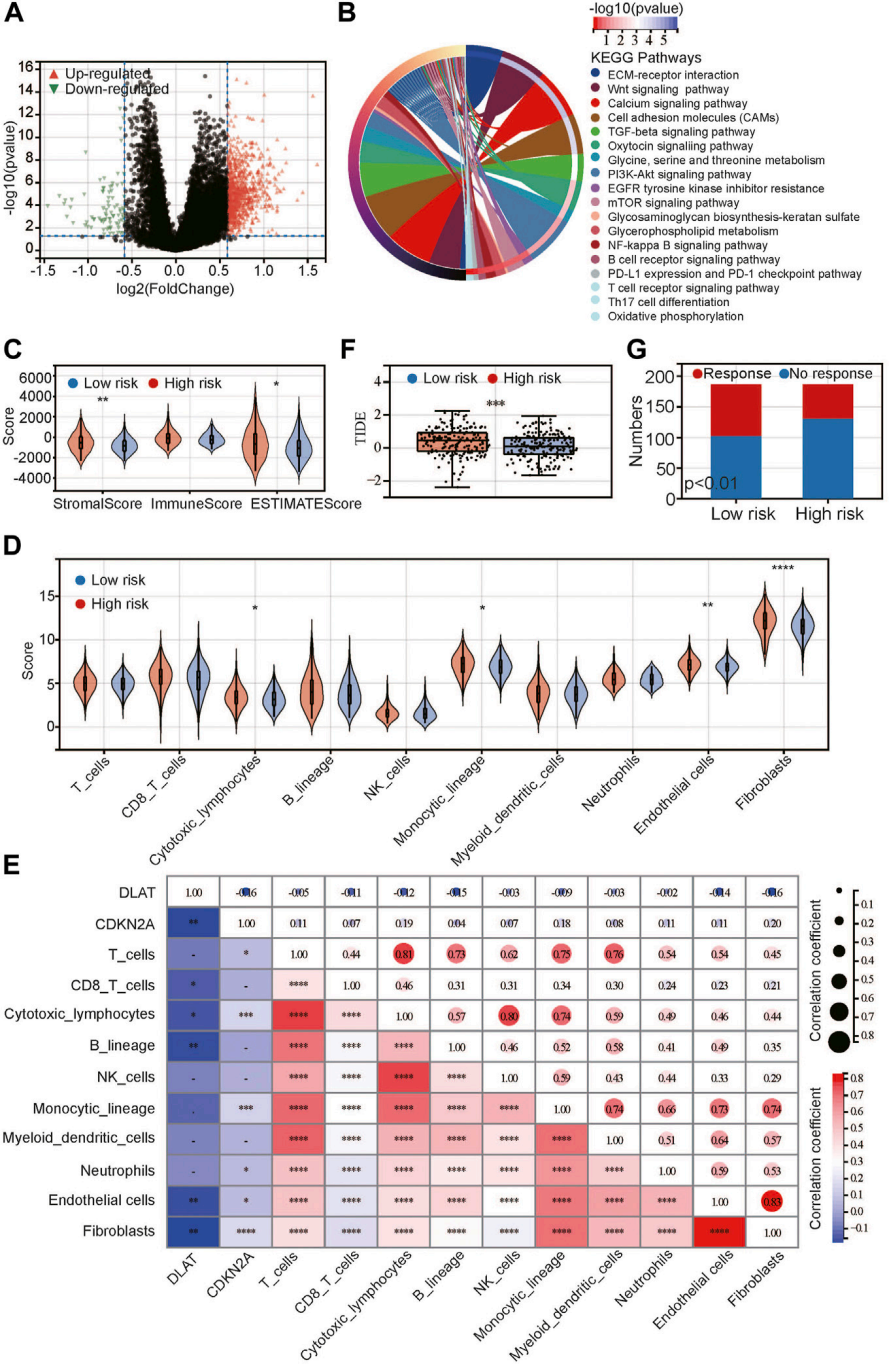
Relationship between risk score and immune/stromal infiltration. **(A)** Volcano plot of differentially expressed genes; **(B)** KEGG pathway enrichment between low- and high-risk groups; **(C**,**D)** Difference between low- and high-risk groups by ESTIMATE **(C)** and MCP-Counter **(D)** algorithms; **(E)** Correlation between CRGs and immune-related scores; **(F)** Difference between low- and high-risk groups in TIDE score; **(G)** Predicted response for immunotherapy. *p < 0.05, **p < 0.01, ***p < 0.001, ****p < 0.0001.

To identify the potential differences between the high-risk and low-risk groups, we conducted differential expression analysis. The DEGs between the high-risk and low-risk groups were identified (Figure 5A), of which there were more upregulated DEGs than downregulated DEGs. The heatmap shows the top 50 DEGs with fold change >2. The expression of these genes was significantly different between the low- and high-risk groups. KEGG pathways (Figure 5B) were enriched in several classical tumor signaling pathways, including the Wnt signaling pathway, PI3K Akt signaling pathway, mTOR signaling pathway, etc. In addition, we found that the immune-related signaling pathways included the B-cell and T-cell receptor signaling pathways, Th17 differentiation, and the PDL1 and PDK1 checkpoint signaling pathways. We speculated that immune infiltration may be closely related to tumor prognosis.

Based on the above results, we used the ESTIMATE algorithm to evaluate the proportion of immune, stromal and tumor cells in tumor tissues (Figure 5C). The results showed that there was no difference in the immune score between the high-risk and low-risk groups, but the stromal score and ESTIMATE score were significantly higher in the high-risk group, indicating that high-risk tumors had more stromal cells and tumor cells. Next, we used the MCP-Counter algorithm to further evaluate immune cells and stromal cells (Figure 5D). The results showed that stromal cells, including endothelial cells and fibroblasts, were significantly more abundant in the high-risk group than in the low-risk group. In addition, most immune cells did not differ between the two groups, except that cytotoxic lymphocytes and monocytes were higher in the high-risk group than in the low-risk group. We analyzed the correlations between DLAT and CDKN2A and the above scores and found that DLAT was negatively correlated with the above scores, while CDKN2A was positively correlated with the above scores (Figure 5E). Both DLAT and CDKN2A are related to endothelial cells and fibroblasts. Additionally, DLAT was mainly related to CD8 T cells, cytotoxic lymphocytes and B cells, while CDKN2A was mainly related to T cells, cytotoxic cells, monocytes and neutrophils. Next, we analyzed the TIDE score between the high-risk and low-risk groups to evaluate the immune escape ability (Figure 5E). The results showed that the TIDE score in the high-risk group was significantly higher than that in the low-risk group, and we predicted the response to immunotherapy of the two groups (Figure 5F). The response rate in the high-risk group was significantly lower than that in the low-risk group. This may be attributed to the difference in immune escape ability.

## Discussion

In the current study, the expression patterns of 10 CRGs in different clinical characteristics of CRC were explored. Then, we analyzed the relationship between CRGs and the prognosis of CRC. Two CRGs, DLAT and CDKN2A, were found to be closely related to OS in CRC. A risk score based on these two genes was established. Functional enrichment analysis showed that immune-related pathways were enriched between the low- and high-risk groups. Finally, we found that the high-risk group was associated with higher immune escape and more interstitial cells.

A recent study has shown that cuproptosis is a new form of cell death (Tsvetkov et al., 2022). Under normal physiological conditions, copper regulates energy metabolism depending on the fatty acylation of the members of the tricarboxylic acid cycle (Tsvetkov et al., 2022). In previous studies, copper was found to be higher in tumor tissues than in normal tissues (Ishida et al., 2013). The reprogramming of energy metabolism in tumor tissue leads to a reduction in energy metabolism, which makes the tumor not completely dependent on the energy provided by mitochondria (Carles-Fontana et al., 2022). The imbalance of copper homeostasis in the physiological state will lead to copper death (Tsvetkov et al., 2022). These results suggest that there may be a significant imbalance in the genes related to copper death in tumors so that tumor cells can avoid copper death. In this study, compared with normal tissues, there were 8 copper death-related genes in tumors with obvious imbalance, and the expression trend of most genes had a positive or negative relationship with pathological stages. These results suggest that the imbalance of copper death genes may be closely related to the survival and progression of CRC.

In further analysis, we found that DLAT and CDKN2A were closely related to the prognosis of patients with CRC, and then we established a risk score. In the training set and validation set, we confirmed that the risk score has robust predictive performance. A previous study reported that the glycolysis-related gene DLAT was associated with the prognosis of colon cancer (Chen et al., 2020). In addition, DLAT, as a CRG, was used to predict the prognosis of clear cell renal cell carcinoma (Bian et al., 2022). For CDKN2A, it has been reported that CDKN2A induces epithelial–mesenchymal transformation and promotes the metastasis of CRC (Shi et al., 2022). Therefore, CDKN2A has been used as a prognostic marker of CRC (Kang et al., 2022). In our study, DLAT and CDKN2A were combined to predict the prognosis of CRC and performed well.

It is well known that the level of immune cells is closely related to the prognosis of tumors (Zhang et al., 2022). In this study, we found that the levels of immune cells and stromal cells were different in CRC patients with high and low risk scores. In terms of immune cells, we found that high-risk patients have higher cytotoxic lymphocytes and monocytes, higher immune cell escape ability and a low immune response rate. Previous studies have reported that cytotoxic lymphocytes and monocytes are important components of maintaining the tumor immune environment and help kill tumor cells (Shalapour and Karin, 2021). However, in our study, the high-risk group may have a higher immune escape ability; therefore, although the proportion of cytotoxic lymphocytes and monocytes was high, the high-risk group had a poor effect on immunotherapy overall. Next, we found that DLAT and CDKN2A were significantly associated with a variety of immune cells. Similar to our study, it has reported that CDKN2A are related to the immune response and tumor immune microenvironment in tumors (Sun et al., 2020). In addition, we found that stromal cell levels, including endothelial cells and fibroblasts, in high-risk tumors were significantly higher than those in low-risk tumors. Endothelial cells and fibroblasts are important cells that constitute the tumor microenvironment and can promote the occurrence and development of tumors (Mun et al., 2022). The poor prognosis of high-risk tumors may also be related to the increase in these cells in the tumor microenvironment.

In this study, we analyzed the relationship between CRGs and clinical characteristics of CRC in detail, and constructed a prognosis model and risk score for CRC based on CRGs. We also clarified the relationship between risk score and immune microenvironment in CRC. However, there are still some limitations to point out. First, the identification of relationship between CRG expression and the clinical features of CRC was based on CRG mRNA from RNA-seq data in TCGA. It is necessary to further detect the CRG protein expression difference between different clinical features; 2. Second, the construction and validation of the prognosis model of CRC were conducted in two TCGA datasets from the American population. External cohorts from other countries are needed to validate the model; 3. The relationship between risk score and immune microenvironment was identified by bioinformatic analysis and needs further experimental validation.

In conclusion, our study systematically summarized the expression patterns of CRGs in different clinical characteristics of CRC. It was confirmed that some CRGs are closely related to the prognosis of CRC. The risk score based on CRGs showed good performance in predicting prognosis. Finally, we found that the risk score was related to the level of immune infiltration and the level of interstitial cells.

## Data Availability

The original contributions presented in the study are included in the article/Supplementary Material, further inquiries can be directed to the corresponding author.

## Supplementary material

Supplementary Figure S1 Additional CRGs-related analysis in colorectal cancer. **(A)** Principal component analysis between normal and tumor samples in TCGA-colorectal cancer dataset; **(B)** CRG expression in different recurrence status; **(C)** CRG expression in different M stage; **(D)** Risk plots showed relationship between risk score, prognosis and gene expression; **(E)** Heatmap showed gene expression between low- and high-risk groups.

Supplementary Figure S2 Additional relationship between CRGs and clinical characteristics of CRC. **(A**–**C)** CRG expression in different status of lymph node metastasis **(A)**, T stage **(B)** and pathological stages **(C)** of adenocarcinoma; **(D**–**F)** CRG expression in different status of lymph node metastasis **(D)**, T stage **(E)** and pathological stages **(F)** of mucinous adenocarcinoma.
