# Supplementary figures and images for: Cuproptosis-Related genes in the prognosis of colorectal cancer and their correlation with the tumor microenvironment
Source: Front Genet. 2022 Sep 28;13:984158. doi: 10.3389/fgene.2022.984158 (PMC9554006; doi:10.3389/fgene.2022.984158)

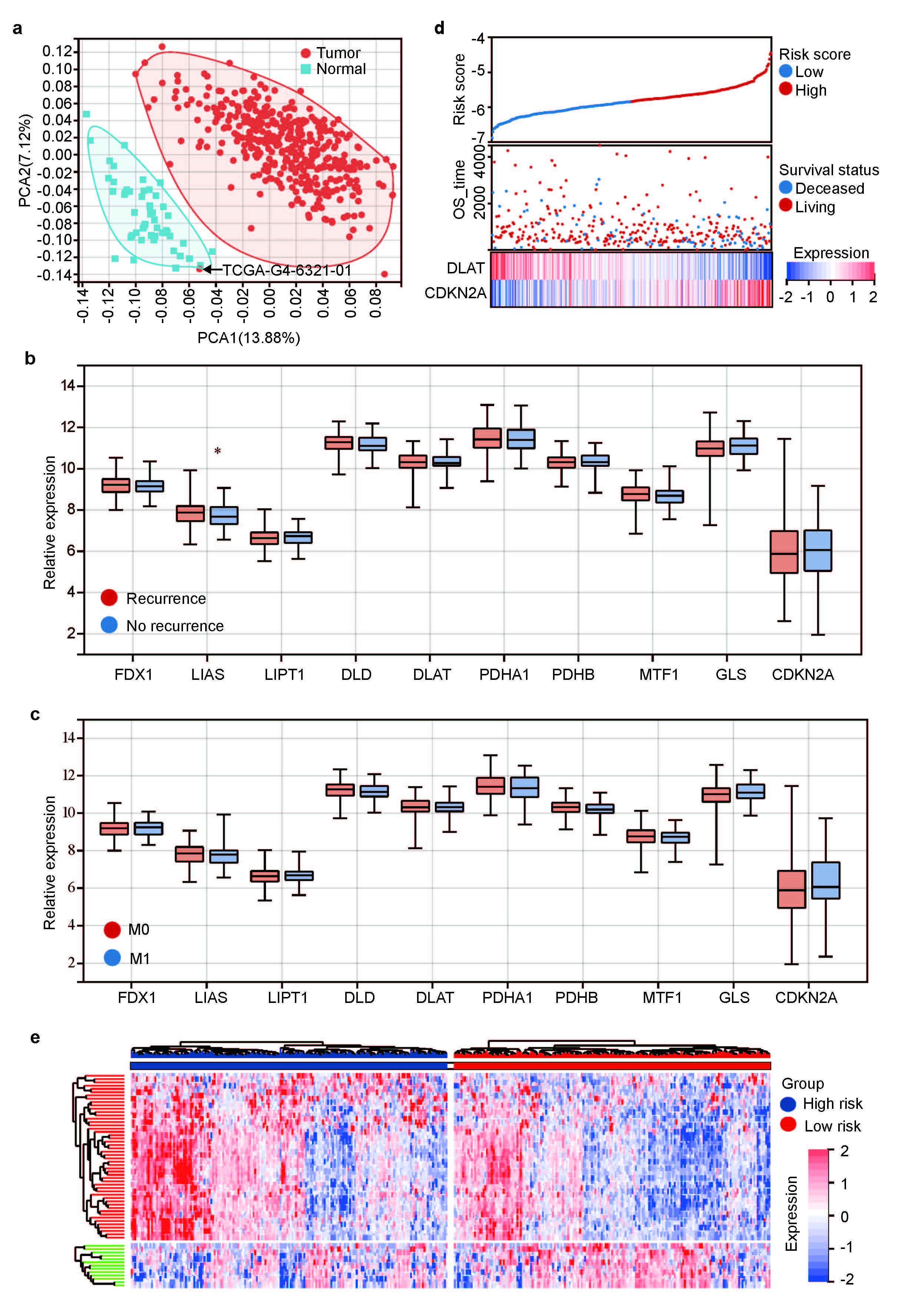

Supplement: Supplementary file 1 [file Image1.JPEG]

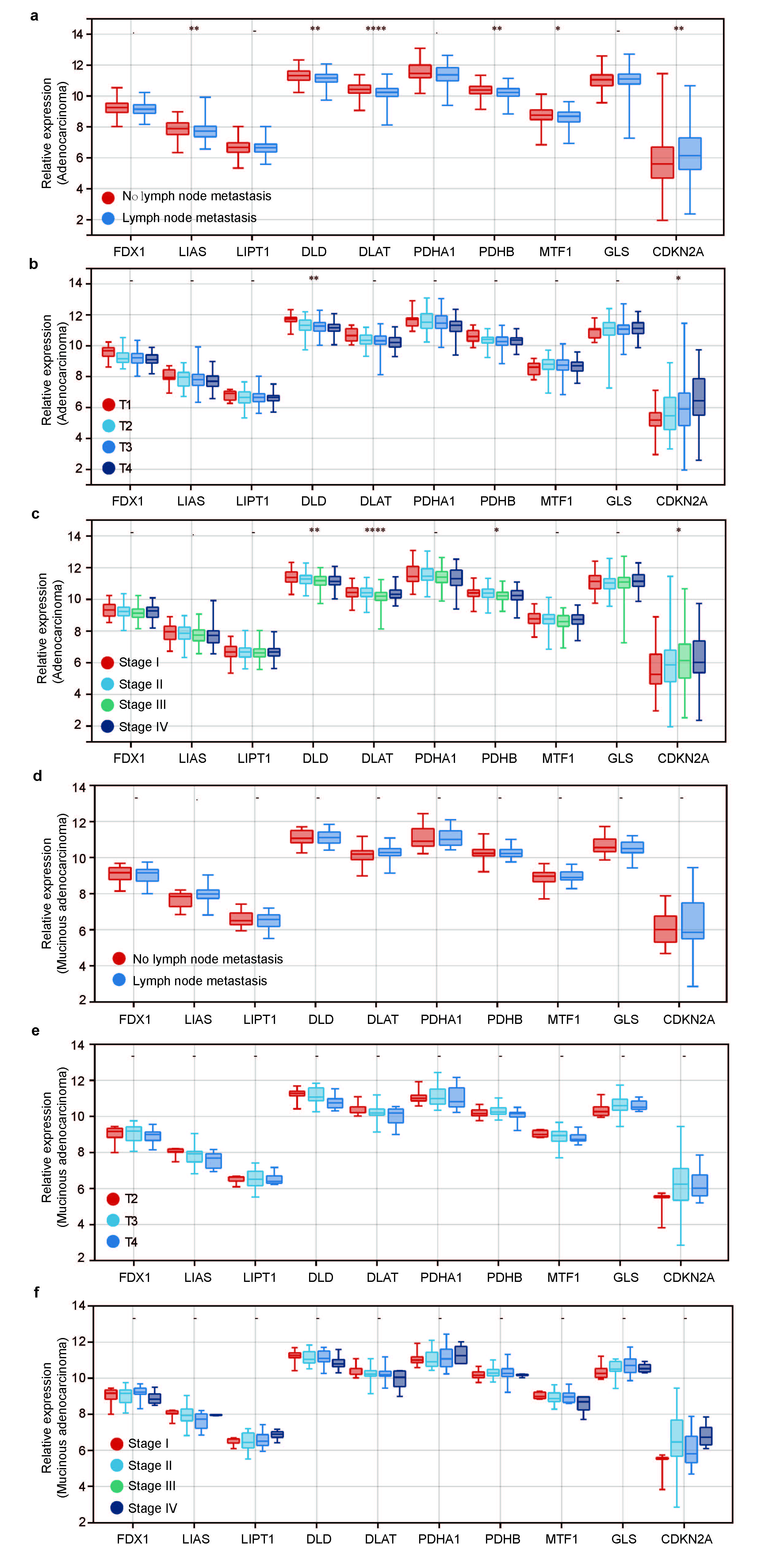

Supplement: Supplementary file 2 [file Image2.JPEG]
