# Supplementary material for: Cuproptosis-Related genes in the prognosis of colorectal cancer and their correlation with the tumor microenvironment
Source: Front Genet. 2022 Sep 28;13:984158. doi: 10.3389/fgene.2022.984158 (PMC9554006; doi:10.3389/fgene.2022.984158)
Supplement: Supplementary file 3 [file Table1.DOC]

| **Table S1 multivariate Cox regression analysis for OS in colorectal cancer** | | | | | |
| --- | --- | --- | --- | --- | --- |
| **Variables** | **OR** | **SE** | **p** | **95% CI** | |
|  |  |  |  | **Lower** | **Higher** |
| Age | 0.405 | 0.297 | 0.002 | 0.226 | 0.724 |
| T |  |  | 0.000 |  |  |
| refer to T1 |  |  |  |  |  |
| T2 | 1.439 | 1.375 | 0.791 | 0.097 | 21.285 |
| T3 | 0.821 | 0.777 | 0.800 | 0.179 | 3.77 |
| T4 | 0.266 | 0.317 | 0.000 | 0.143 | 0.496 |
| Stage |  |  | 0.000 |  |  |
| refer to Stage I |  |  |  |  |  |
| Stage II | 0.07 | 0.926 | 0.004 | 0.011 | 0.432 |
| Stage III | 0.224 | 0.343 | 0.000 | 0.115 | 0.44 |
| Stage IV | 0.406 | 0.327 | 0.006 | 0.214 | 0.771 |
| Riskscore | 2.237 | 0.289 | 0.005 | 1.269 | 3.943 |
| Abbreviations: OR, Odd ratios; SE, Standard error; CI, Confidence interval | | | | |  |
|  |  |  |  |  |  |
